# A Practical Approach to Identifying Processed White Meat of Guinea Fowl, Rabbit, and Selected Fish Species Using End-Point PCR

**DOI:** 10.1155/2021/7710462

**Published:** 2021-07-22

**Authors:** Anita Spychaj, Kamila Goderska, Emilia Fornal, Magdalena Montowska

**Affiliations:** ^1^Department of Meat Technology, Poznań University of Life Sciences, ul. Wojska Polskiego 31, 60-624 Poznań, Poland; ^2^Department of Food Technology of Plant Origin, Poznań University of Life Sciences, ul. Wojska Polskiego 31, 60-624 Poznań, Poland; ^3^Department of Pathophysiology, Medical University of Lublin, ul. Jaczewskiego 8b, 20-090 Lublin, Poland

## Abstract

Among the foodstuff, most often adulterated are white meat and meat products as well as fish and fish products. For this reason, we evaluated in practice the possibilities of identifying selected species of white meat, i.e., guinea fowl and rabbit as well as four fish species, namely, pollock, hake, sole, and panga, in thermally treated samples. The aim was to check whether the previously published in the scientific literature species-specific primers allows for the identification of processed meat using the end-point PCR technique. To identify the six species, the short sequence fragments (from 130 to 255 bp) of 12S rRNA, COX3, mitochondrial ATP synthase Fo subunit 6 (ATP6) gene, pantophysin (Pan I) gene, 5S rRNA gene, and microsatellite markers (locus: Phy01-KUL) were selected. Stability and specificity of the six pair primers were evaluated on cooked and autoclaved meat, and commercially processed food samples such as rabbit and guinea pâtés, ready-made baby food, and breaded, fried, and deep-frozen fish products. The method proved to be useful for the authentication of severely processed food products against fraudulent species substitution and mislabelling and this approach may be an alternative to more advanced and more expensive PCR techniques.

## 1. Introduction

The development of modern technologies related to the acquisition of raw materials, their processing, and transport has enabled the distribution of food products throughout the world. This is conducive to expanding the market and satisfying the ever-growing demand for food. However, in an era of globalised trade, the complex supply chain of foodstuffs creates many opportunities for food fraud. The pressure to offer ever cheaper food means that it is not always produced fairly, as evidenced by numerous recent fraudulent practices publicised in mass media, such as adding melamine to milk powder, adding Sudan dyes to meat, and replacing beef with horse meat [1]. Among the types of violation notified in the Administrative Assistance and Cooperation System (AAC), available to the EU Member States, mislabelling (68.17%) came first, followed by unapproved treatment and/or process (15.35%), documents (8.93%), and replacement/dilution/addition/removal in products (7.55%) [2, 3]. Meat, fish, and their products are among the most often adulterated foodstuffs. Meat with high market value, attractive for nutritional reasons and desirable sensory features, is particularly vulnerable to adulteration. The white meat species that arouse interest among customers and are susceptible to economically motivated adulterations include rabbit, guinea fowl, and fish.

In Europe, rabbit meat is especially appreciated in the Mediterranean countries for culinary reasons as well as due to its nutritional value and low allergenic potential; rabbit is recommended for children and adolescents [4, 5]. Rabbit meat is characterised by a high content of protein (approx. 22%), unsaturated fatty acids (approx. 60.5% of all fatty acids, including 32.5% polyunsaturated fatty acids), and potassium; it is also a good source of B vitamins, especially vitamin B12 [4, 6]. On the European market, guinea fowl meat is particularly recognised in France and Italy, and sensory features similar to those of venison make this species of meat also appreciated in the United States [7]. Guinea fowl meat contains favourable proportions of potassium to sodium and is a source of polyunsaturated fatty acids, accounting for about 29% of all total fatty acids [8, 9]. Fish, especially of marine origin, is the recommended component of a balanced diet. Fish meat contains protein with high biological value and polyunsaturated fatty acids, mainly n-3, and is rich in vitamins and minerals; thus, the regular consumption of fish reduces the risk of heart disease and strengthens the skeletal system [10, 11].

Food adulteration can have serious negative effects on public health and economics and may also hinder the maintenance of personal beliefs related to one's outlook on life or religion, since a large part of the world's population excludes certain types of meat from their daily diet. As mentioned above, fraudulent practices are most often associated with the substitution or mislabelling of food components and may mislead consumers, exposing them to harmful substances. Some meat proteins, for instance, actin, myoglobin, parvalbumins, tropomyosin, serum albumin, and primarily those of fish origin, can cause allergic reactions when ingested [12, 13]. The threat to human health may be toxins, whose presence can be found in fish and seafood. Neurotoxins accumulate in muscle tissue as a result of environmental degradation. Consumers are also concerned about the possibility of consuming meat from animals that may have suffered from diseases such as avian influenza, African swine fever (ASF), foot and mouth disease (FMD), and bovine spongiform encephalopathy (BSE); therefore also for those reasons, the buyer should be guaranteed that they buy a product that is verified in terms of composition [14].

Another issue that is widely discussed nowadays is the degradation of nature through intensified agriculture, the production of food of animal origin, and the overexploitation of natural resources, which contribute to global warming. Excessive exploitation of the environment, in the case of seafood, is associated, among others, with overfishing or obtaining endangered species, which, in consequence, may lead to the destruction of animal populations. Unfair food production and unfair trade cause economic losses not only for competing enterprises but also for consumers themselves, who may feel deceived when purchasing a product that does not meet their expectations. Detection of unfair practices by control bodies undermines the trust of customers not only to a specific manufacturer but often to the entire industry involved in the production of a given product.

Previously in the literature, raw and thawed meat of selected fish species, including gadoid and Aegean fish species (e.g., hake, pollock, and sole), was identified using isoelectric focusing and immunological methods by detecting species-specific enzymes present in the fraction of sarcoplasmic proteins in the obtained electrophoretic separations [15, 16]. Fish species were also identified by genetic methods, e.g., raw pollock meat, hake, and sole meat, raw and heated at 100°C for 15 min, as well as hake in raw meat, semiprocessed, and processed products using the PCR-RFLP method [17–20]. Conventional PCR and PCR-RFLP analysis of a 12S rRNA gene fragment was used to differentiate between sole and Greenland halibut [21, 22] while DNA barcoding of cytochrome oxidase-I sequence was applied to authenticate raw meat and processed panga products in Italy and Vietnam [23, 24]. Pollock and hake were also identified in various fish products by combining DNA barcoding and PCR-RFLP methods [25, 26].

Processed meat and fish products are particularly vulnerable to adulteration because the morphological features of the raw material are removed during the production, and this prevents the use of visual methods to verify the composition of the final ready-to-eat product. Besides, the meat of individual species may have a similar texture after processing. For this reason, advanced methods based on protein analysis, e.g., electrophoretic and mass spectrometry-based techniques, or DNA analysis, are used to identify the species of meat present in processed food products. At present, well-established methods for meat speciation are based on PCR; however, a lower efficiency of the PCR method was reported for highly processed samples due to thermal denaturation and degradation of the fragments of nucleic acids monitored, problems with the extraction of genetic material, and cross-reactivity between species [27, 28]. For instance, the quality of extracted DNA (i.e., yield and integrity) from bovine supraspinatus muscle was affected by microwave cooking, which was observed for both mitochondrial and nuclear regions [27]. In another study, boiling and baking in a dry oven affected species determination and quantification of chicken, pork, and beef mitochondrial DNA (mtDNA) using real-time PCR [28].

Since meat speciation in ready meals using PCR is affected by thermal conditions, the present study was undertaken to verify the possibility of identification of selected white meat species in severely processed and complex products by genetic methods. The aim was to investigate whether heat treatment would affect the stability of the selected markers and whether the thermally processed meat of guinea fowl, rabbit, and fish species such as hake, pollock, panga, and Dover sole could be identified based on specific DNA sequence fragments. Additionally, cross-species analyses were carried out, and commercially processed pâtés and ready meals were examined to verify if processing and the presence of additional ingredients in the sample impede the detection of specific meat types. In this study, the end-point PCR technique and primers available in the scientific literature were used to evaluate the identifiability of processed white meat of the selected species. The end-point PCR is easy to use and less expensive compared to more advanced techniques such as rt-PCR, sequencing, or mass spectrometry; thus, it may be implemented in the routine screening of processed products.

## 2. Materials and Methods

### 2.1. Samples

The research material was obtained from the following animal species: guinea fowl (*Numida meleagris*), rabbit (*Oryctolagus cuniculus* f. *domesticus*), Dover sole/common sole (*Solea solea*), hake (*Merluccius merluccius*), panga catfish (*Pangasianodon hypophthalmus*), and Alaska pollock (*Gadus chalcogrammus*). The DNA from other commonly consumed species was also isolated to exclude possible cross-reactions, i.e., chicken (*Gallus gallus*), turkey (*Meleagris gallopavo*), goose (*Anser anser*), sheep (*Ovis aries*), pig (*Sus scrofa*), and cattle (*Bos taurus*). Fish (fresh Dover sole and frozen hake, panga, and pollock) and fresh meat of farmed animals were purchased at local stores and supermarkets. Commercially processed products of various manufacturers were purchased at local stores in Poland, such as pâtés, ready-made baby food, and, in the case of fish, breaded, fried, and deep-frozen products.  Table 1 presents the species composition and processing types of the tested products. Immediately after the purchase, samples of about 5 g were cut off under sterile conditions, transferred to sterile tubes, and stored at −20°C until further DNA analysis.

### 2.2. Heat Processing

Fish and meat slices with a thickness of up to 25 mm were wrapped in aluminium foil and heated in a Rational Combi convection oven model SCC 61 (Landsberg am Lech, Germany). Heating was carried out at a temperature of 160°C with an air humidity in the oven chamber of 75% until reaching a core temperature of 75°C. The same set of samples was sterilised at 121°C at a pressure of 0.1 MPa for 20 min. Samples were cooled, transferred to sterile tubes, and stored at −20°C until further DNA analysis.

### 2.3. DNA Isolation, Concentration, and Purity

The DNA was isolated from all samples in duplicate using the PureLink™ Genomic DNA Mini Kit (Invitrogen, Carlsbad, CA, USA) according to the protocol supplied by the manufacturer, with slight changes. The concentration of the extracted DNA was determined by measuring UV absorbance at 260 nm (A260) and 280 nm (A280), using a NanoDrop™ OneC microvolume UV-Vis spectrophotometer (Thermo Fisher Scientific Inc., Waltham, MA, USA).

For instrument calibration, TE buffer (10 mM Tris-HCL, 0.1 mM EDTA) was used, and DNA purity was estimated by calculating the A260/A280 ratios. Samples with A260/A280 ratios in the range of 1.8–2.0 were considered high-quality pure samples, free from protein, RNA, and other contaminants. The integrity of the DNA form raw and heated meat tissues was checked by 2% agarose (Sigma-Aldrich, Schnelldorf, Germany) gel electrophoresis containing SimplySafe™ stain (EURx Ltd., Gdańsk Poland) in 1x TBE buffer (tris-borate-EDTA). Electrophoresis was performed in a SUB15 gel unit (Hoefer, Inc., Holliston, MA) at 150 V for 45 min.

### 2.4. PCR Amplification

Six species-specific primer pairs were selected for the amplification of guinea fowl, rabbit, hake, panga, pollock, and sole DNAs. Another six primer pairs specific to chicken, turkey, goose, pig, cattle, and sheep were used to control cross-reactions. Primers were custom-synthesised by TIB MOLBIOL GmbH (Berlin, Germany); the primer sequences and methods of sample processing are shown in Table 2. Reaction conditions were applied in accordance with given references in Table 2, except for rabbit and pollock primer pairs, for which the reaction conditions were tested empirically.

Favourable conditions for the formation of an amplification product from rabbit DNA by conventional PCR were as follows: an initial heat denaturation step at 95°C for 5 min, followed by 35 cycles consisting of 95°C for 30 s for DNA denaturation, 56°C for 30 s for primer annealing, and 72°C for 45 s for DNA extension. The final elongation step was carried out at 72°C for 5 min. In the case of pollock, the PCR reaction consisted of 45 consecutive cycles: 95°C for 10 s, 65°C for 20 s, and 72°C for 60 s, with a final elongation step at 72°C for 10 min. Amplification of species-specific fragments was carried out in reaction mixture consisting of 100 ng DNA (in the case of pollock and fish products, 20 ng DNA were collected), 1 *μ*M of each primer, 2.5 *μ*L of 10x PCR buffer (Sigma-Aldrich), 0.2 mM dNTP (Sigma-Aldrich), and 0.25 *μ*L (5 U/*μ*L) DNA polymerase (Sigma-Aldrich). The reaction solution was made up to a final volume of 25 *μ*L with sterile water for molecular biology (Merck Millipore, Darmstadt, Germany). Amplification was performed in an S1000 Thermal Cycler (Bio-Rad Laboratories, Inc., Hercules, CA, USA).

### 2.5. Separation and Visualisation of the PCR Products

Amplicons were separated by horizontal electrophoresis in 2% agarose gel (Sigma-Aldrich, Schnelldorf, Germany) containing SimplySafe™ stain (EURx Ltd., Gdańsk Poland) in 1x TBE buffer (tris-borate-EDTA). Electrophoresis was performed in a SUB15 gel unit (Hoefer, Inc., Holliston, MA) at 150 V for 45 min. The molecular size of the PCR products was verified using a PCR Low Ladder (Sigma-Aldrich), and the resulting DNA fragments were visualised by UV transillumination and analysed using a Gel Doc™ XR+ System (Bio-Rad Laboratories, Inc., Hercules, CA, USA).

## 3. Results and Discussion

### 3.1. Primer Selection

We focused on the practical ability to identify specific species of white meat (guinea fowl and rabbit) and fish (pollock, hake, sole, and panga) using selected genetic markers. The markers species specificity and stability in thermally processed meat samples were evaluated first; subsequently, we determined whether the matrix complexity of ready meals, i.e., the presence of vegetables, spices, and additives in the analysed sample, impedes their detection. To identify guinea fowl, rabbit, pollock, hake, sole, and panga meat, the sequence fragments of 12S rRNA, COX3, mitochondrial ATP synthase Fo subunit 6 (ATP6) gene, pantophysin (Pan I) gene, 5S rRNA gene, and microsatellite markers (Locus: Phy01-KUL) were selected (Table 2). The primers have been designed previously for animal species identification in the food and forensic science field, but, apart from guinea fowl, they have not been tested on samples subjected to heat treatment. Therefore, in the present work, the six pair primers' stability and specificity were evaluated on cooked, autoclaved, and commercially processed food samples.

For genetic identification, both mitochondrial (mtDNA) and nuclear DNA sequences are used. The mtDNA sequences are characterised by low intraspecies variability and high interspecies variability and numerous point mutations; they can also occur in a greater number of copies compared to nuclear DNA. These features mean that mtDNA enables the differentiation of even closely related species and can be obtained more easily compared to genomic DNA, also from severely heat-treated samples [38, 39]. In the present study, three of the six primer sequences, namely, COX3, 12S RNA, and mt ATP synthase Fo subunit 6 gene, specific to rabbit, guinea fowl, and pollock, respectively, were sequences associated with mtDNA.

Unlike mitochondrial DNA, nuclear DNA is more difficult to obtain from animal cells because it only occurs in two copies in the cell nucleus. Only about 25% of the genomic DNA constitutes genes and gene-related sequences. A significant part of it, approximately 70-80%, is nongenic DNA, of which about 20-30% correspond to moderately or repeatedly distributed sequences scattered throughout the genome or united and arranged in large arrays of tandemly repeating sequences. Arrayed sequences include satellite DNA, which is divided into three classes: satellite, minisatellite, and microsatellite.

In an attempt to identify panga, microsatellite markers were used at the Phy01-KUL locus (Table 2). The polymorphism of microsatellites is based on the variable number of short tandem repeats (STRs) of the same nucleotide motif [40]. In the concerted evolution process, these sequences become species-specific [41]. The methods based on dispersed and repeating units use the fact of their repeated appearance in the genome, which increases the pool of the molecular target studied [42].

The 5S rRNA and pantophysin I (Pan I) gene sequences used in this study are present in genomic DNA. The 5S rRNA gene consists of a highly conserved 120-nucleotide coding sequence that occurs in the genome in the form of tandem repeats. These repeats are separated by a nontranscribed sequence (NTS) whose length and sequence are species-specific [31]. The Pan I gene applied in this work is also a highly polymorphic marker. Pantophysin is an integral membrane protein found in small cytoplasmatic microvesicles that are thought to have a function in a variety of intracellular shuttling pathways [17]. Previously, pantophysin I gene has been used especially to characterise the genetic diversity of Atlantic cod [43–45].

### 3.2. Impact of Processing on DNA Extraction and Quality

The basic issue in genetic methods, such as PCR, is the appropriate quality and quantity of genetic material, which, after isolation from the sample, is subjected to further analysis. These parameters are determined, among others, by the extraction method used, the sample composition, and the type of processing [46, 47]. During the extraction of genetic material from meat products, a number of compounds may pass into the DNA preparation and affect its purity, potentially inhibiting the PCR reaction. These compounds include fat, polysaccharides, milk proteins, glycogen, collagen, iron, cobalt, Mallard reaction products, and phenolic compounds. The inhibitor may also be genetic material of bacterial, plant, or animal origin present in the sample, other than the target DNA to be analysed [48, 49], as well as the remainder of the reagents used for DNA extraction. Reaction inhibitors interfere with the PCR amplification process, among others, by reducing or completely inhibiting DNA polymerase activity [46, 50].

When assessing DNA purity, the UV light absorption ratio A260/A280 is in the range of 1.8–2.0 for DNA extracts free of impurities [51]. In our work, we obtained good-quality DNA isolates from all analysed samples, i.e., from frozen and thawed, cooked, and sterilised muscle tissue and commercial products, and the A260/A280 ratios for the analysed samples were within the recommended limits. However, we observed that DNA preparations isolated from pollock, hake, panga, and commercial fish products contained less DNA compared to preparations from Dover sole and other types of meat. This is in line with previous reports where the muscle tissue of fish, with a few exceptions, usually contained less DNA compared to the tissue of cattle, pigs, or poultry [52]. A freezing factor could also affect the lower efficiency of isolation of fish genetic material, since in the case of three analysed fish species, i.e., pollock, hake, and panga, frozen fish fillets were used for the study. Long-distance transport of frozen products, as it is often the case with fish or seafood, and all operations related to their distribution can contribute to temperature fluctuations inside the load, consequently, to DNA degradation. Previous studies on beef and earthworms have shown that even a single thawing and refreezing cycle of the product led to DNA degradation [51, 53].

In our research, from 91.7% of heat-treated samples, more genetic material was obtained than from raw/thawed tissue, as measurements showed a higher DNA concentration. These results coincide with the results obtained by another author [54], who observed a significant impact of heating methods on the quality and quantity of DNA obtained from meat. This author showed that the amount of DNA isolated from raw meat compared to meat grilled at 300°C or heated in a microwave oven at 560 W for 5 min was significantly lower, which might be a result of the destruction of nuclear or cell membranes due to heating, promoting the release of more DNA from cells. However, even though the preparations obtained from heated samples have a higher concentration of DNA, also due to the loss of water, in general, this material is of lower quality, i.e., more fragmented compared to that present in raw or frozen/thawed samples [54].

### 3.3. Identification of Rabbit and Guinea Fowl Species

**Figure 1:**
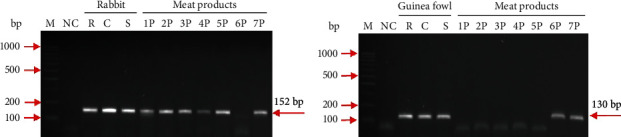
Identification of rabbit (a) and guinea fowl (b) PCR-amplified products in heated meat and meat products. Lanes: M: ladder marker; NC: negative control; R: raw meat; C: cooked meat; S: sterilized meat; 1P: rabbit pâté (sterilised); 2P: rabbit pâté (sterilised); 3P: ready-made baby dish (sterilised); 4P: ready-made baby dish (sterilised); 5P: rabbit pâté (roasted, home-made); 6P: pâté with guinea fowl (sterilised); 7P: rabbit and guinea fowl pâté (roasted, home-made).

Our research concerned the identification of meat heated in different conditions, causing DNA degradation. Therefore, PCR primers selected for the study were amplifying relatively short sequence fragments, i.e., from 130 bp to 255 bp (Table 2). In the first stage, rabbit and guinea fowl raw/thawed, cooked, and autoclaved meats were analysed and, when the specificity and stability of the amplified fragments were confirmed, species identification was performed in the processed products. Species-specific PCR primer sets amplified 152 bp rabbit and 130 bp guinea fowl products. These amplified PCR products of the expected sizes were detected in raw meat samples, cooked in a convection oven (to obtain a core temperature of 75°C), and even sterilised at 121°C for 20 min. The identification of rabbit and guinea fowl amplified products in thermally processed meat is shown in Figures 1(a) and 1(b).

Cross-reactions were also carried out to verify the species specificity of the selected primers. For primers designed to detect rabbit DNA, cross-reactions were carried out with pig, cattle, and sheep DNA, while guinea fowl primers were cross-tested with chicken, turkey, and goose DNA. The obtained results proved that the markers selected for the identification of these two species were species-specific (data not shown).

Although DNA is thought to be more resistant to temperature or high pressure than other compounds, it is also affected and subjected to degradation [55, 56]. Similarly, low pH, UV radiation, and high humidity can cause chemical modification of a DNA molecule, manifesting in its fragmentation or the formation of PCR artifacts [49]. It is also likely that enzymes present in carbohydrate-rich products, such as soy preparations, lead to DNA degradation [56].

The use of degraded DNA for PCR may cause a reaction background. It is also difficult to amplify longer DNA sequences, and sometimes, it is completely impossible due to the degradation of the target material [49, 54]. In previously published studies, it was not possible to amplify a 439 bp product from horse meat heated at 120°C for 30 min [57], a product of 835 bp from pork meat sterilised at 121°C at 15 psi pressure for 20 min [38], and a short product of 271 bp that could not be obtained from beef fried for 80 min when the temperature of the fat exceeded 190°C [58]. In another study, the amplification of a product > 800 bp from beef heated above 95°C failed [59].

In the present work, the detection of rabbit and guinea fowl PCR-amplified products in sterilised meats, as well as the analysis of processed products containing these two tested species, was successful; of the seven products investigated, two were roasted and five sterilised. The rabbit was identified in both pâtés and ready-made baby food. Species detection in processed products, where the examined matrix is severely degraded due to thermal treatment, and its composition is complex. Baby dishes were complex products with a high degree of processing to ensure microbiological safety; thus, the production of such food includes cooking, comminution, and sterilisation; they also contained, in addition to meat, various vegetables, cereals, and vegetable oils (Table 1).

Rabbit meat declared at 8.5 and 8% in baby food (samples 3P and 4P, respectively) was detected (Figure 1(a)). Guinea fowl was correctly identified at the declared amounts of 5 and 11.2% (samples 6P and 7P; Figure 1(b)). The successful identification of the two white meat species in the sterilised products confirmed that the selected markers are species-specific and stable, regardless of the treatment that the processed products had undergone. Due to the amplification of short sequence fragments, i.e., 152 bp and 130 bp, it was possible to detect specific DNA, which was fragmented due to the technological processes carried out.

### 3.4. Identification of Hake, Pollock, Panga, and Sole Species

We focused on identifying processed white meat derived from three fish species that are widely available in the form of frozen fillets and ready meals and are popular among consumers in Europe, i.e., Alaska pollock, hake, and panga catfish. In addition, we analysed common sole/Dover sole, a more expensive species, valued by consumers and offered in restaurant dishes and therefore susceptible to adulteration with cheaper species. Confirming the ability to identify fish species in processed samples is important for honest food manufacturers, restaurateurs, and consumers, because there is also a risk that cheaper species such as hake, pollock, and panga can replace more expensive species in processed foodstuffs.

**Figure 2:**
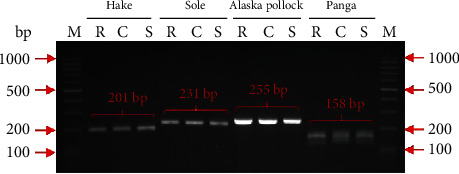
Identification of PCR-amplified products specific to selected fish species in thermally treated meat samples. Lanes: M: ladder marker; R: raw fish meat; C: cooked fish meat; S: sterilized fish meat.

At the initial stage of the study, the PCR primer pair that was chosen to detect Alaska pollock DNA amplified a 492 bp product (data not shown). However, no amplification product of the selected sequence fragment was obtained with its use both in raw and cooked pollock samples, indicating significant DNA degradation in frozen pollock fillets. For this reason, the four primer pairs selected to identify all fish species to be analysed amplified shorter PCR products, ranging from 158 to 255 bp (Table 2). Thus, in the second attempt, to identify pollock DNA, primers intended to amplify a 255 bp product were applied. It turned out that a product of this size can be amplified from raw/frozen fish but also from the samples subjected to cooking and autoclaving at 121°C for 20 min (Figure 2). In the case of hake, sole, and panga, when PCR products of 201, 231, and 158 bp were amplified, respectively, their presence was confirmed in all three types of samples, i.e., the nonheated and thermally processed fish tissue. However, significant differences were observed in the intensity of the bands of the amplified products between the four species (Figure 2). This indicates different levels of DNA degradation between the fish species studied, probably due to different storage conditions and storage times maintained by distributors and retailers. Nevertheless, detection of guinea fowl, rabbit, and fish PCR-amplified products in all processed samples was successful, which confirmed that the selected markers are species-specific and stable regardless of the treatment that the processed products had undergone.

**Figure 3:**
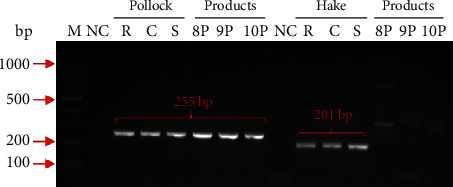
Identification of Alaska pollock and hake in processed products. Lanes: M: ladder marker; NC: negative control; R: raw fish; C: cooked fish; S: sterilized fish; 8P: breaded pollock fillet (fried, deep-frozen); 9P: white fish fillet (fried, deep-frozen); 10P: breaded pollock fillet (fried, deep-frozen).

The ability to identify selected fish species in thermally processed food products available commercially was verified. The examined products were breaded fish fingers, which, in the production process, are prefried and then sold deep-frozen. The manufacturers declared pollock meat solely in their composition. The PCR primers specific to Alaska pollock DNA enabled the identification of this species in all analysed samples, but when using hake-specific primers, some nonspecific products were observed, i.e., bands of low intensity and different sizes (Figure 3). Such nonspecific products were not detected for sole and panga primer pairs. The results demonstrate the stability of the selected genetic markers and the possibility of their practical use to study the species composition of commercially produced products using the conventional PCR method. However, we observed significant DNA degradation in deep-frozen fish fillets and fish products.

Processed products distributed through a long and complex supply chain are exposed to unfair counterfeiting practices. Although there are some legal regulations, international and intrastate, aimed at eliminating illegal practices, food frauds are constantly detected. To date, many studies and methods of genetic identification of species of fish and farm animals have been published, but still, a few of them concerned complex and severely processed food products such as sterilised pâtés, ready-made baby dishes, and ready meals. Previously, Alaska pollock has been identified among samples (fillets) collected from department stores in Tehran using DNA barcoding based on a 650 bp fragment of the mitochondrial cytochrome oxidase subunit I gene (COI) [60]. Three Alaska pollock samples were labelled incorrectly.

Recently, a DNA microarray assay for the authentication of 10 fish species, among them Alaska pollock (*Gadus chalcogrammus*), common sole (Solea solea), and striped catfish (*Pangasianodon hypophthalmus*), has been designed based on 16S rDNA and cyt b probe sequences [61]. The microarray was tested on fresh, frozen, smoked, and marinated, but no thermally processed samples. The PCR-RFLP and sequencing of mt cyt b were developed for intraspecies differentiation of hake samples on the Czech market, but the results were not always conclusive [62]. In another study, fish dishes sold in commercial restaurants and sushi bars in Brussels were collected for mislabelling detection using DNA barcoding and cytochrome oxidase subunit I (COI) gene [63]. Sole was mislabelled in 11.1% samples, and other species mislabelled regularly were cod (13.1%) and bluefin tuna (95%). However, most modern genetic methods are still more expensive and more time-consuming and require further data processing, making them less suitable for routine species identification purposes compared to conventional PCR analysis.

## 4. Conclusions

Our research confirmed the species specificity of selected genetic markers and their heat resistance during cooking and sterilisation. The identification of selected white meat species, namely, rabbit, guinea fowl, Alaska pollock, hake, common sole, and panga, can be performed in samples exposed to high temperatures and in processed products of complex composition. The method can be useful for the authentication of severely processed food products against fraudulent species substitution and mislabelling. However, we observed that DNA preparations isolated from frozen pollock, hake, panga, and commercial fish products contained less DNA compared to fresh samples of other meats and products, and we were not able to detect longer DNA-amplified products of 492 bp, specific to Alaska pollock, which indicates the significant DNA degradation in deep-frozen fish fillets and fish products.

## Figures and Tables

**Table 1:** Processing type and declared species composition of the meat products tested.

Sample No.	Product	Species declared	Product composition
1P	Rabbit pâté (sterilised)	Pig, rabbit, chicken	Pork jowls, rabbit meat 21%, chicken liver, milk, eggs, salt, Armagnac 0.8%, spices, natural flavours
2P	Rabbit pâté (sterilised)	Pig, rabbit	Pork meat, rabbit meat 25%, pork liver, white wine, Herbes de Provence 0.3%, hazelnut flour, spices, flavours
3P	Ready-made baby dish (sterilised)	Rabbit	Water, boiled rice 23%, broccoli 20%, rabbit meat 8.5%, rapeseed oil 2%
4P	Ready-made baby dish (sterilised)	Rabbit	Carrots 44%, water, parsley 15%, rabbit meat 8%, white grape juice, potatoes, corn starch, vegetable oils (rapeseed, sunflower), dill 0.15%. Vegetables total 64.7%
5P	Rabbit pâté (roasted, home-made)	Rabbit, chicken, pig	Rabbit meat, chicken liver, pork liver, pork eggs, breadcrumbs, vegetables, salt, black pepper
6P	Pâté with guinea fowl (sterilised)	Chicken, pig, guinea fowl	Chicken meat 21%, water, pork fat, chicken skins, pork skins, chicken liver 6%, pork liver 6%, guinea fowl meat 5%, semolina (from wheat), salt, modified starch, pea fibre, emulsifier E472c, spices, rice flour, aromas (contain celery), dried tomatoes, corn protein hydrolysate, preservative E250
7P	Rabbit and guinea fowl pâté (roasted, home-made)	Pig, rabbit, guinea fowl, chicken	Pork 24%, pork liver 13.9%, rabbit meat 11.2%, guinea fowl 10.2%, chicken liver 6.3%, eggs, breadcrumbs, vegetables, salt, black pepper
8P	Breaded pollock fillet (fried, deep-frozen)	Pollock	Pollock fillet, breading dough 12% (5% wheat flour, 5% eggs, salt, black pepper), wheat flour, sunflower oil, salt, yeast
9P	White fish fillet (fried, deep-frozen)	Pollock	White fish fillet 61% (pollock), breadcrumbs (wheat flour, salt, red pepper, yeast), sunflower oil, water, wheat flour, potato starch, salt
10P	Breaded pollock fillet (fried, deep-frozen)	Pollock	Pollock fillet 65%, wheat flour, water, sunflower oil, potato starch, salt, yeast, spices

**Table 2:** Species-specific PCR primers and methods of sample processing in accordance with cited reference.

Species	Sequence (5′→3′)	Product size (bp)	GenBank accession No.	Molecular target	References	Processing method
Guinea fowl	ACCTCAAAACAATCTTAGCCACCA TTCTCAGGCGGATACTTAGGTATTG	130	AM902518	12S rRNA	[29]	Raw meat, pasteurization (72°C, 30 min), sterilisation (121°C, 20 min)
Rabbit	GACCTCCAACAGGCATCA GGTAATGGCTAGAGCTTGCTG	152	NC001913	COX3	[30]	Meat, bone, blood, hair, saliva, testicular and ovarian tissue
Dover sole	GCACACGTTAACGTCCTTCT GGGGACACAGACTCCTTTTATAG	231	HQ681116	5S ribosomal RNA gene	[31]	Raw meat
Hake	CCCGTTTAGGTTTGTGGTTGC TGTGCAGACGAACTGTGGTTC	201	EU301630	Pantophysin (Pan I) gene	[32]	Raw, chilled, or frozen fish
Panga	GTAAACAGAGCCACCTGCGG CAGATCCACACCCACAACACC	158	AJ131380	Microsatellite markers	[33]	No information
Alaska pollock	GATTTATCGCCCGCTTTACTAAC TGCCTTCTGGGAGGAAGTGG	255	AB094061	Mitochondrial ATP synthase Fo subunit 6 (ATP6) gene	[34]	Raw meat, roe
Chicken	GGGACACCCTCCCCCTTAATGACA GGAGGGCTGGAAGAAGGAGTG	266	X52392	Mitochondrial genome	[35]	Meat and bone meal (MBM), cooking (100°C, 2 h), sterilisation (133°C, 20 min)
Turkey	GCATTCTCTTCTGTGGCCT GAGGGTGAGAAGTAAGAC	185	L08381.1	Mitochondrion cytochrome b gene	[36]	Egg and egg powder
Goose	ACAGGACATACCCTAACA GTCCAGGCTTAGATTGTG	387	EU571959	D-loop	[37]	Raw meat, cooking (100°C, 15 min), sterilisation (121°C, 15 min)
Sheep	GACCTCCAACAGGCATCA GGTAATGGCTAGAGCTTGCTG	225	AF039171	Cytochrome oxidase subunit 2 (COII) gene	[35]	Meat and bone meal (MBM), cooking (100°C, 2 h), sterilisation (133°C, 20 min)
Pig	GCCTAAATCTCCCCTCAATGGTA ATGAAAGAGGCAAATAGATTTTCG	212	AF039170	Cytochrome oxidase subunit 2 (COII) gene	[35]	Meat and bone meal (MBM), cooking (100°C, 2 h), sterilisation (133°C, 20 min)
Cattle	GCCATATACTCTCCTTGGTGACA GTAGGCTTGGGAATAGTACGA	271	V00654.1	Mitochondrial genome	[35]	Meat and bone meal (MBM), cooking (100°C, 2 h), sterilisation (133°C, 20 min)

## Data Availability

The data used and/or analysed in the study are available from the corresponding author on reasonable request.
